# Tackling cardiovascular co-morbidities in HIV-positive patients: who, how and where?

**DOI:** 10.7448/IAS.17.4.19725

**Published:** 2014-11-02

**Authors:** Sophie Rolls, Emma Denneny, Rebecca Marcus, Rebecca O'Connell

**Affiliations:** Newham University Hospital, Barts Health NHS Trust, HIV Medicine, London, UK

## Abstract

**Introduction:**

Cardiovascular disease (CVD) is a significant cause of non-AIDS-related morbidity and mortality in HIV-positive individuals [1]. Management of CVD and associated risk factors in HIV are complicated by drug interactions [2]. Optimal management can require specialist input. A previous cohort review highlighted CVD, comorbidity and cardiovascular (CV) risk in our patients [3]. In response, a combined HIV and cardiovascular monthly clinic was established: an HIV consultant works in real time with a cardiologist. The clinic manages CV disease, complex CV co-morbidities e.g. refractory hypertension, hyperlipidaemia, and assesses primary prevention. A dietician works alongside the clinic.

**Aims:**

Describe the clinic caseload; record clinic interventions and outcomes; recommend service development.

**Materials and Methods:**

We conducted a retrospective notes review of patients attending the co-morbidity clinic from January 2012 to May 2014. Data collected: demographic, HIV, CVD, CV risk, investigations and clinical interventions.

**Results:**

From a cohort of approximately 960 patients (70% African), 60 (6%) were seen in the co-morbidity clinic over the specified time period. Median age was 53 (range 24-80). Although 60% of our cohort is female, 43% (26/60) of the CVD clinic were female. 42 (70%) were African. The mean CD4 was 560 (range 48-1339). All patients were on ART and 6 (10%) had a detectable viral load > 400 copies/mL. Clinic caseload: i) CVD: 9 had a prior CV event (ACS or CVA); 5 had CCF; new diagnoses included LVH (2), cardiac dysfunction (6); AF (2); atrial thrombus (1). ii) Co-morbidities: 48(80%) had hypertension – 10 (16.6%) were on quadruple therapy; 17 (28%) had diabetes; 35 (58%) were on a statin. Three had their smoking status clearly documented. Seventeen (28%) were referred to the dietician. Investigations included echo, 24-hour BP/ tape, CT angio, cardiac MR.

**Conclusions:**

The joint clinic facilitated real-time decision making on clinical interventions. Patient access to cardiac investigations was expedited. Patients attended fewer outpatient appointments. Both cardiology and HIV clinicians preferred the benefits of joint working. Clinical outcomes were difficult to assess and will need further definition. Recommendations for development include: improved CV risk assessment, improved outcome measures, links to smoking cessation services.
